# Patient knowledge, home care practices, and peri-implant disease: a preliminary survey analysis of awareness gaps and cleaning behaviors

**DOI:** 10.3389/froh.2026.1826003

**Published:** 2026-06-15

**Authors:** Sandra Stuhr, Suncica Travan, Ann M. Decker

**Affiliations:** Department of Periodontics and Oral Medicine, School of Dentistry, University of Michigan, Ann Arbor, MI, United States

**Keywords:** dental implant, interdental aids, oral biofilm, oral hygiene techniques, oral irrigators, peri-implantitis, plaque removal, primary prevention

## Abstract

**Introduction:**

Dental implants longevity depends on proper maintenance and home care. Understanding patients' knowledge, practices, and barriers to optimal care is essential for developing effective educational strategies and improving long-term implant outcomes.

**Materials and methods:**

This cross-sectional study evaluated adult patients with at least one restored dental implant at the University of Michigan School of Dentistry. Peri-implant health status was determined using pre-established diagnostic criteria. Participants completed a 13-item survey assessing home care behaviors (cleaning frequency, duration, methods, confidence, challenges) and knowledge regarding implant infection risk and systemic links. Associations between survey variables and disease status were analyzed using Fisher's exact test and chi-square tests (*p* < 0.05).

**Results:**

Thirty-four subjects (17 males, 17 females) and 71 implants were assessed (20 peri-implant health, 43 peri-implant mucositis, 8 peri-implantitis). Despite 88.2% reporting daily cleaning, disease prevalence was 70.4%. At the implant level, retention method (*p* = 0.011), and plaque index (*p* < 0.001) were significantly associated with disease. At the patient level, all peri-implantitis patients had a history of periodontitis (Fisher's exact *p* = 0.029). Facial keratinized mucosa width (KMW) did not show a significant association with peri-implant diagnosis, although lingual KMW did between peri-implant health and peri-implantitis (*p* = 0.01), and peri-implantitis and peri-implant mucositis (*p* = 0.04). Thin mucosal tissue showed a non-significant trend toward association with more severe disease, progressing from health to peri-implantitis (*p* = 0.172). Cleaning frequency was the strongest predictor (*p* = 0.035) of peri-implant health. Nearly one-third of patients received no professional implant care instruction. Interdental brushes were least utilized (8.8%), while string floss was most common (82.4%). Knowledge regarding implant disease showed no significant associations with disease status. Most patients reported moderate to high confidence regarding their home care abilities, regardless of disease status (*p* = 0.22).

**Discussion:**

High disease prevalence despite daily home care and confidence levels reveals a critical gap between patient effort and effective prevention. Cement-retained restorations emerged as the strongest implant-level risk factor. Nearly one-third lacking professional instruction and minimal interdental brush adoption suggests targets for intervention. Future research should evaluate whether targeted educational interventions emphasizing infection risk and proper cleaning techniques can improve both patient knowledge and clinical outcomes, focusing on primary prevention of peri-implant disease.

## 1 Introduction

Dental implants are widely used to replace missing teeth and are associated with high long-term survival and patient satisfaction (1). As the number of implants placed continues to rise, attention has increasingly shifted from short-term osseointegration to long-term maintenance of peri-implant tissues (2). Unlike natural teeth, implants are not as resistant to inflammatory disease, and their longevity depends on sustained biofilm control, consistent professional monitoring, and patients' ability to perform effective daily home care (2).

**Figure 1:**
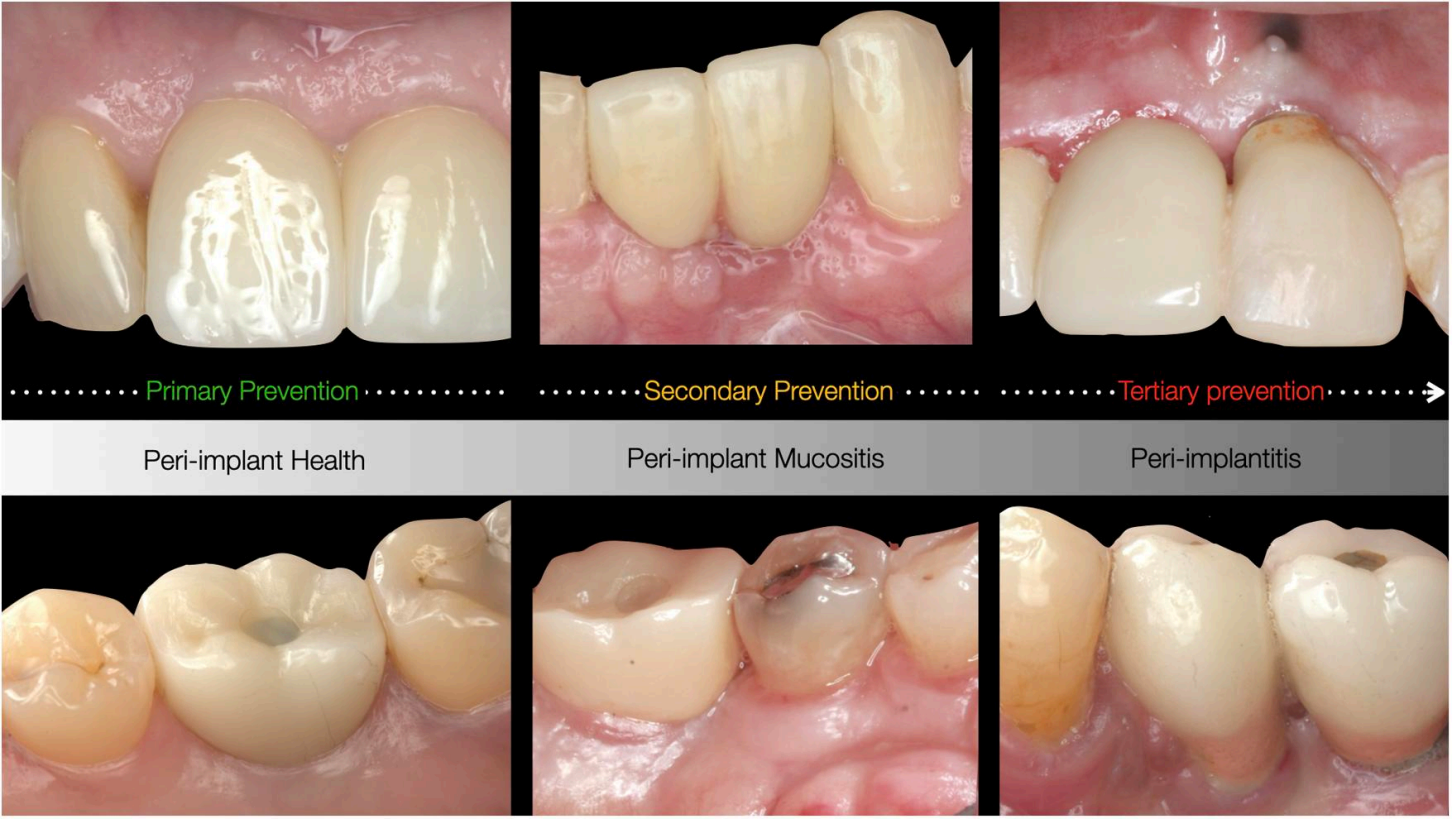
A combination of clinical photographs demonstrating examples of peri-implant health, peri-implant mucositis, and peri-implantitis, and the progression from primary to tertiary prevention in the context of peri-implant disease.

Peri-implant diseases, specifically peri-implant mucositis and peri-implantitis, represent the most common biologic complications affecting implant therapy (2). Peri-implant mucositis is characterized by plaque-associated inflammation of the mucosa surrounding the implant and is considered reversible with adequate plaque control, while peri-implantitis involves inflammation accompanied by progressive supporting bone loss and may threaten implant survival (3–5). Established and suspected risk factors and indicators for peri-implant disease include inadequate plaque removal, a history of periodontitis, tobacco use, and systemic or behavioral factors that can influence immune response and self-care (3). Because peri-implant mucositis can progress to peri-implantitis in susceptible individuals, early identification and prevention are central goals of implant maintenance programs (3). Primordial, primary, secondary, and tertiary prevention of peri-implant diseases have been more recently described in the literature in an attempt to address risk assessment and risk factor control in the context of preventing disease occurrence and recurrence, starting even before implant placement (Figure 1) (5–7). In the context of implant home care, the focus on primary prevention of peri-implant disease is paramount, as this involves maintaining a healthy implant that has not yet developed clinical signs of disease.

Although professional maintenance is important, daily patient-performed plaque control is the foundation of prevention (3). Effective home care around implants often requires additional interdental cleaning methods (e.g., interdental brushes, floss, oral irrigators) and technique modifications to accommodate implant restorations and prosthetic contours (3). However, patients may not receive consistent implant-specific instruction, may be unaware that implants can develop infections, or may face practical barriers such as dexterity limitations, discomfort, recall of instructions, or time constraints (8–11). While the published evidence on peri-implant diseases has exponentially increased over the last decade, current literature on the primary prevention of implant diseases and conditions is notably scarce, hindering the clinicians' abilities to provide evidence-based recommendations.

Assessing patients' knowledge, behaviors, confidence, and perceived barriers, alongside clinical measures of peri-implant health, can help identify modifiable gaps that contribute to disease risk. Therefore, the purpose of this cross-sectional study was to evaluate peri-implant tissue status in a university-based dental clinic population and to examine associations between peri-implant diagnoses and patient-reported home care practices, sources of instruction, and key elements of implant-related knowledge. Findings from this work can inform targeted, practical educational strategies aimed at improving long-term peri-implant health outcomes.

## 2 Materials and methods

This study was a cross-sectional survey approved by the University of Michigan's Institutional Review Board for human research (HUM#00272580). All subjects provided informed consent prior to enrollment in the study. This study adhered to the Checklist for Reporting of Survey Studies (Supplementary File 1) (12).

### 2.1 Study population

Adults seeking dental treatment at predoctoral clinics of the University of Michigan from February 2025 through July 2025 were screened to participate in our study. To be eligible for inclusion, patients had to be 18 years or older with at least one restored dental implant and scheduled for a comprehensive oral examination or periodic oral examination. The patient recruitment design was via convenience sampling due to the exploratory nature of this study.

### 2.2 Clinical assessment

To classify and analyze patient behaviors and knowledge related to dental implant maintenance, the following demographic and clinical variables were collected: age, sex, history of periodontitis, education level, years of implant function, implant location, number of implants, restoration type (single unit, fixed dental prosthesis), retention type (cement vs. screw-retained), probing depth (PD, six sites/implant), bleeding on probing (BOP, six sites per implant) (13), plaque index (14), keratinized mucosa width (KMW, buccal/facial and lingual), and soft tissue phenotype (thin/thick based on probe visualization) (15, 16). Clinical photographs of each dental implant restoration were also taken (buccal/facial and lingual/palatal aspects).

Peri-implant diagnoses (peri-implant health, peri-implant mucositis, or peri-implantitis) were made based on the most recent AAP/EFP World Workshop on Peri-Implant Diseases and Conditions and reported on both an implant and patient level (4). Two clinical examiners, both board-certified periodontists, ensured consistency based on 5 implants (2 maxillary, 3 mandibular within the same patient not involved in the study) in terms of clinical measurements by obtaining an ICC of 0.83 for PD, 0.73 for BOP, 0.80 for PI, 0.90 for KMW, and 0.97 for soft tissue phenotype (S.S., A.D.). Furthermore, all members of the research team (S.S., A.D., S.T.) confirmed the peri-implant diagnosis for each implant based on overall consensus.

### 2.3 Questionnaire

A paper-based 13-question survey (Supplementary File 2) was distributed to 3 faculty attendings familiar with the study design supervising the pre-doctoral clinics (S.S., A.D., S.T.). After obtaining written informed consent, eligible subjects were asked to complete the anonymized questionnaire, which included topics related to their implant home care, general knowledge regarding peri-implant diseases, the type of instruction provided regarding implant home care, risk factors, and their perception of their peri-implant health status. The questionnaire consisted of multiple-choice and Likert-scale questions divided into 3 general sections: behavioral practices, knowledge and awareness, and psychosocial factors.

Cleaning frequency (Question 1) was measured on a 4-point ordinal scale (1 = “Rarely/Never”, 2 = “Occasionally”, 3 = “Every other day”, 4 = “Daily”). Cleaning methods (Question 4) captured all applicable methods used, including manual toothbrush, powered toothbrush, floss, interdental aids, oral irrigation, and oral mouthrinses. Confidence level of performing oral hygiene methods was assessed on a 5-point scale (1 = “Not confident” through 5 = “Extremely confident”). Knowledge items (Questions 10–12) assessed awareness of peri-implant infection risks, systemic health connections, and relationship to periodontal disease on hierarchical scales ranging from “not aware” to “fully understand”. Education level was categorized as: less than high school, high school diploma, some college, or college degree or higher. Stress level over the past month was measured on a 10-point scale groups into 5 categories from minimal (0–2) to severe (9–10) stress.

Binary variables were created for the following specific behaviors: received professional instructions, uses interdental brush, uses any interproximal cleaning method, and cleans daily. Examiners ensured that all surveys were complete and were available to answer any questions if clarification was needed. All completed surveys were assigned a study ID number, did not contain any identifiable patient information and were kept in a locked cabinet.

### 2.4 Statistical analysis

Survey responses were merged with clinical examination data at the patient level. For patients with multiple implants, the worst diagnosis among a patient's implants was assigned as the patient-level diagnosis for the purpose of group comparisons in demographic and survey data. Clinical parameters were assessed at the implant level, as each implant was independently examined and diagnosed. Descriptive statistics were assessed for the total sample. Count and frequencies were calculated for categorical responses. Mean and standard deviation were calculated for each continuous clinical measurement.

Bivariate tests with continuous measures were performed using Spearman rank correlation for associations between the ordinal survey responses and clinical parameters, and Mann–Whitney or Kruskal–Wallis tests for group comparisons for non-parametric data. For categorical measures, Chi square and Fisher's exact tests were used for hypothesis testing, with statistical significance defined as *p* < 0.05 for primary comparisons, and *p* < 0.01 for other comparisons. All statistical analyses were performed using R (Version 4.5.2). Although this was a pilot survey, collected data on established risk factors and indicators was used to assess potential confounding. Univariate and stratified analyses were conducted to examine any associations between these factors and peri-implant disease status, and to assess whether these factors confounded any primary associations.

## 3 Results

### 3.1 Study demographic characteristics

Thirty-five patients completed both the survey and clinical examination, representing a 100% response rate among eligible participants. One subject (1 male with peri-implantitis) withdrew from the study after completion, leaving 34 final subjects available for analysis. The mean age of subjects was 64.5 ± 11.2 years (range 46–86 years), with 50% being male and 50% female. Fifty percent of subjects had a documented history of periodontitis. All peri-implantitis patients were male (*p* = 0.089) and had a history of periodontitis (chi-square *p* = 0.056; Fisher exact peri-implantitis vs. other groups, *p* = 0.029). None of the associated risk factors or indicators showed significant association with peri-implant disease status. Data related to patient demographics are shown in Table 1.

**Table 1:** Demographic characteristics, patient-level.

Parameter	Peri-implant health (*n* = 6)	peri-implant mucositis (*n* = 24)	Peri-implantitis (*n* = 4)	*p*-value
Age, years	62.7 ± 11.2	67.0 ± 10.2	65.8 ± 11.2	0.669
Gender				
Male, *n* (%)	2 (33.3)	11 (45.8)	4 (100.0)	0.09*
Female, *n* (%)	4 (66.7)	13 (54.2)	0 (0.0)	
History of Periodontitis				
Yes, *n* (%)	2 (33.3)	9 (37.5)	4 (100.0)	0.056
No, *n* (%)	4 (66.7)	15 (62.5)	0 (0.0)	
Smoking				
Never smokers, *n* (%)	4 (66.7)	15 (62.5)	2 (50.0)	0.58
Former smokers, *n* (%)	1 (16.7)	5 (20.8)	0 (0.0)	
Current smokers, *n* (%)	1 (16.7)	4 (16.7)	2 (50.0)	
Diabetes				
Yes, *n* (%)	1 (16.7)	2 (8.3)	0 (0.0)	0.65
No, *n* (%)	5 (83.3)	22 (91.7)	4 (100.0)	
Alcohol Consumption				
Yes, *n* (%)	2 (33.3)	11 (45.8)	0 (0.0)	0.21
No, *n* (%)	4 (66.7)	13 (54.2)	4 (100.0)	
Osteoporosis				
Yes, *n* (%)	0 (0.0)	5 (20.8)	0 (0.0)	0.30
No, *n* (%)	6 (100.0)	19 (79.2)	4 (100.0)	
Education Level				
College degree or higher, *n* (%)	5 (83.3)	13 (54.2)	0 (0.0)	0.09*
Some college, *n* (%)	0 (0.0)	9 (37.5)	3 (75.0)	
High school or less, *n* (%)	1 (16.7)	2 (8.3)	1 (25.0)	
Stress Level (past month)				
Minimal (0–2)	0 (0.0%)	4 (16.7%)	2 (50.0%)	0.28
Mild (3–4)	2 (33.3%)	9 (37.5%)	0 (0.0%)	
Moderate (5–6)	3 (50.0%)	6 (25.0%)	1 (25.0%)	
High (7–8)	1 (16.7%)	4 (16.7%)	0 (0.0%)	
Severe (9–10)	0 (0.0%)	1 (4.2%)	1 (25.0%)	

* *p* <0.05.

### 3.2 Clinical characteristics

Mean PD differed significantly based on peri-implant diagnosis, along with plaque index values and BOP scores. Mean PD were 2.98 ± 0.93 mm for implants diagnosed with peri-implant health, 3.80 ± 0.77 mm with peri-implant mucositis, and 5.60 ± 1.37 mm with peri-implantitis (*p* < 0.001).

Total BOP score and the number of BOP-positive sites were both statistically significantly associated with peri-implant diagnosis (*p* < 0.001). The mean plaque index was 0.57 ± 0.51, 1.17 ± 0.73, and 1.75 ± 0.89 for implants diagnosed with health, peri-implant mucositis, and peri-implantitis, respectively (*p* < 0.001).

Facial or buccal KMW did not differ significantly between groups (*p* = 0.167). After adjustment for smoking, the association also did not reach significance (*p* = 0.25). However, pairwise comparisons between healthy implants and those with peri-implantitis, and between peri-implant mucositis and peri-implantitis, demonstrated a significant difference for lingual KMW (*p* = 0.01 and 0.04, respectively). Thus, decreased lingual KMW was associated with increased severity of peri-implant disease. Specific details related to clinical parameters in relation to peri-implant diagnosis are shown in Table 2.

**Table 2:** Clinical parameters in relation to peri-implant diagnosis and pairwise comparisons (PD, probing depth; BOP, bleeding on probing; KMW, keratinized mucosa width) at the implant-level.

*n*	Peri-implant Health	Peri-implant Mucositis	Peri-implantitis	*p*-value
	21	42	8	
Mean PD, mm (Mean ± SD)	2.98 ± 0.93	3.80 ± 0.77	5.60 ± 1.37	<0.001*
Adjusted for smoking				<0.001*
Adjusted for diabetes				<0.001*
Adjusted for alcohol				<0.001*
Adjusted for osteoporosis				<0.001*
Maximum PD, mm(Mean ± SD)	3.71 ± 1.27	4.52 ± 0.97	6.88 ± 1.64	<0.001*
Adjusted for smoking				<0.001*
Adjusted for diabetes				<0.001*
Adjusted for alcohol				<0.001*
Adjusted for osteoporosis				<0.001*
BOP Total Score (Mean ± SD)	0.24 ± 0.54	3.76 ± 2.35	4.50 ± 1.93	<0.001*
Adjusted for smoking				<0.001*
Adjusted for diabetes				<0.001*
Adjusted for alcohol				<0.001*
Adjusted for osteoporosis				<0.001*
# of BOP + Sites(Mean ± SD)	0.24 ± 0.54	2.98 ± 1.58	3.75 ± 1.39	<0.001*
Adjusted for smoking				<0.001*
Adjusted for diabetes				<0.001*
Adjusted for alcohol				<0.001*
Adjusted for osteoporosis				<0.001*
Plaque Index (Mean ± SD)	0.57 ± 0.51	1.17 ± 0.73	1.75 ± 0.89	<0.011*
Adjusted for smoking				<0.002*
Adjusted for diabetes				<0.008*
Adjusted for alcohol				<0.009*
Adjusted for osteoporosis				<0.011*
KMW, Buccal, mm (Mean ± SD)	3.00 ± 1.34	2.31 ± 1.37	2.62 ± 1.85	0.167
Adjusted for smoking				0.246
Adjusted for diabetes				0.33
Adjusted for alcohol				0.35
Adjusted for osteoporosis				0.29
KMW, Lingual, mm (Mean ± SD)	3.43 ± 0.98	3.18 ± 1.30	2.00 ± .00	0.056
Adjusted for smoking				0.17
Adjusted for diabetes				0.01*
Adjusted for alcohol				0.18
Adjusted for osteoporosis				0.02*

Pairwise comparisons				
Parameter	PI Health vs. PI Mucositis	PI Health vs. Peri-implantitis	PI Mucositis vs. Peri-implantitis	
Mean PD, mm	0.001*	<.001*	<.001*	
Maximum PD, mm	0.01*	<.001*	<.001*	
Total BOP Score (0–12)	<.001*	<.001*	0.16	
Number of BOP + Sites (0–6)	<.001*	<.001*	0.11	
Plaque Index (0–3)	0.002*	0.001*	0.04*	
KMW, Buccal, mm	0.056	0.410	0.85	
KMW, Lingual, mm	0.732	0.011*	0.04*	

* *p* <0.05.

### 3.3 Implant characteristics

Implant restoration types included a single unit crown, single unit crown and cantilever, adjacent single unit splinted, and fixed dental prosthesis (FDP). Complete breakdown of restoration types and other implant-related factors are displayed in Table 3. The restoration type was not significantly associated with implant disease, although it is important to note that all single unit cantilever and splinted restorations that were cement-retained were diagnosed with peri-implant mucositis or peri-implantitis. Cement-retained restorations were associated with peri-implant mucositis and peri-implantitis (*p* = 0.01), compared to screw-retained restorations.

**Table 3:** Implant-specific parameters in relation to peri-implant diagnosis.

*n*	Peri-implant Health	Peri-implant Mucositis	Peri-implantitis	*p*-value
	21	42	8	
Type of restoration (*n*, %)				
Single unit crown	17 (81.0)	33 (78.6)	7 (87.5)	0.40
Single unit cantilever	0 (0.0)	1 (2.4)	1 (12.5)	
Single unit splinted	0 (0.0)	2 (4.8)	0 (0.0)	
Fixed dental prosthesis	4 (19.0)	6 (14.3)	0 (0.0)	
Retention method (*n*, %)				
Cement-retained	8 (38.1)	24 (57.1)	8 (100.0)	0.01*
Screw-retained	13 (61.9)	18 (42.9)	0 (0.0)	
Implant location (*n*, %)				
Maxillary non-molar	10 (47.6)	14 (33.3	2 (25.0)	0.37
Maxillary molar	3 (14.3)	6 (14.3)	0 (0.0)	
Mandibular non-molar	4 (19.0)	4 (9.5)	2 (25.0)	
Mandibular molar	4 (19.0)	18 (42.9)	4 (50.0)	
Mucosal thickness (*n*, %)				
Thick	16 (76.2)	25 (59.5)	3 (37.5)	0.14
Thin	5 (23.8)	17 (40.5)	5 (62.5)	
Years in function (Mean ± SD)	5.9 ± 4.4	6.2 ± 5.5	10.8 ± 5.0	0.02*

* *p* <0.05.

Implant location was not significantly associated with varying disease statuses (*p* = 0.37) though a trend toward higher disease prevalence among mandibular molar sites was observed.

Mucosal thickness demonstrated a trend, although not statistically significant (*p* = 0.14). Thin mucosa increased with disease severity. In peri-implant health, 23.8% of implants had thin mucosa. In peri-implant mucositis, this value increased to 40.5%, and in peri-implantitis, 62.5%. Furthermore, in all peri-implantitis cases, at least one factor discussed above (cement-retained or thin mucosa) was present, and 62.5% had both. Years in function was significantly associated with peri-implant diagnosis (*p* = 0.02), suggesting that longer implant service time is associated with greater disease severity.

### 3.4 Patient-reported behaviors and home care practices

88.2% of patients reported daily cleaning of their dental implants, with 5.9% cleaning every other day, and 5.9% cleaning occasionally or rarely/never. Cleaning frequency varied significantly based on the peri-implant diagnosis group. All patients with implants diagnosed with peri-implant health reported daily cleaning, compared to 91.7% of those with peri-implant mucositis and 50% of those with peri-implantitis. Thus, cleaning frequency demonstrated a strong negative correlation with clinical parameter. Higher frequency of cleaning was associated with lower mean PD (*p* = 0.0011), lower maximum PD (*p* = 0.0078), and lower plaque index (*p* = 0.0159). It was also significantly associated with peri-implant diagnosis at both the patient level (*p* = 0.02) and at the implant level (*p* < 0.001). However, no significant correlation was found between cleaning duration and any clinical outcome measure.

In relation to home care methods utilized, the most commonly reported cleaning method was dental floss (82.4%), followed by manual toothbrush (55.9%), and powered toothbrush (52.9%). About one-third of patients reported use of an oral irrigator (32.3%), while less frequently used methods included an oral mouthrinse (20.5%) and interdental brush (8.8%). Distribution of cleaning method usage by diagnosis group is presented in Tables 4, 5.

**Table 4:** Oral hygiene methods in relation to peri-implant diagnosis, patient-level.

Hygiene method	PI health (*n* = 6)	PI mucositis (*n* = 24)	Peri-implantitis (*n* = 4)	*p*-value
Manual Toothbrush, *n* (%)	4 (66.7)	13 (54.2)	2 (50.0)	0.832
Powered Toothbrush, *n* (%)	3 (50.0)	14 (58.3)	1 (25.0)	0.460
String Floss, *n* (%)	6 (100.0)	20 (83.3)	2 (50.0)	0.124
Interdental Brush, *n* (%)	0 (0.0)	3 (12.5)	0 (0.0)	0.504
Oral irrigator, *n* (%)	3 (50.0)	7 (29.2)	1 (25.0)	0.588
Oral mouthrinse, *n* (%)	0 (0.0)	5 (20.8)	2 (50.0)	0.159
Daily Cleaning, *n* (%)	6 (100.0)	22 (91.7)	2 (50.0)	0.035*

* *p* <0.05.

**Table 5:** Oral hygiene methods in relation to peri-implant diagnosis, implant-level.

Hygiene method	PI health (*n* = 21)	PI mucositis (*n* = 42)	Peri-implantitis (*n* = 8)	*p*-value
Manual toothbrush, *n* (%)	11 (52.4)	23 (54.8)	4 (50.0)	0.964
Powered toothbrush, *n* (%)	16 (76.2)	24 (57.1)	1 (12.5)	0.010*
String floss, *n* (%)	20 (95.2)	35 (83.3)	2 (25.0)	0.001*
Interdental brush, *n* (%)	1 (4.8)	6 (14.3)	0 (0.0)	0.364
Oral irrigator, *n* (%)	11 (52.4)	12 (28.6)	1 (12.5)	0.069
Oral mouthrinse, *n* (%)	4 (19.0)	11 (26.2)	4 (50.0)	0.261
Daily cleaning	21 (100.0)	38 (90.5)	2 (25.0)	<0.001*

* *p* <0.05.

Patients who used a powered toothbrush had significantly lower plaque index scores compared to non-users (*p* = 0.01). Furthermore, its use was associated with 76.2% of heathy patients, 57.1% of peri-implant mucositis patients, and 12.5% of peri-implantitis patients. Use of string floss was also associated with peri-implant health (*p* = 0.001), with its use associated with 95.2% of healthy patients, 83.3% of peri-implant mucositis patients, and 25% of peri-implantitis patients. Less than 10% of patients reported using interdental brushes, with usage rates being similar across all groups.

Interestingly, oral mouthrinse usage increased with disease severity. 0.0% of patients presenting with peri-implant health, 20.8% with peri-implant mucositis, and 50.0% with peri-implantitis reported its usage. Patients who reported using an oral mouthrinse also had significantly greater mean probing depths compared to non-users (4.41 ± 0.93 mm vs. 3.46 ± 0.93 mm, *p* = 0.016).

### 3.5 Patient-Reported professional instructions and challenges

Two-thirds of patients reported receiving specific instructions on implant home care from their dentist or dental hygienist. However, 32.3% of patients reported receiving no instruction whatsoever. Among patients with peri-implantitis, 50% reported receiving no instruction, compared to 33.3% of those with peri-implant mucositis and 16.7% of those with peri-implant health.

When asked about challenges experienced while performing home care for their dental implants, there was a negative correlation between peri-implant diagnosis and the perception of no significant challenges. In fact, 83.3% of those diagnosed with peri-implant health noted no significant challenges when performing home care, compared to 58.3% of peri-implant mucositis patients and 75% of peri-implantitis patients. None of the patients reported any symptoms related to their dental implant(s), including redness, swelling, drainage or discharge, pain or discomfort, or itching or irritation.

Details regarding instruction sources and perceived challenges are shown in Table 6.

**Table 6:** Patient survey responses regarding confidence, instructions received, and barriers to home care around dental implants.

Confidence in home care	PI Health (*n* = 6)	PI Mucositis (*n* = 24)	Peri-implantitis (*n* = 4)	Total (*N* = 34)	*p*-value
1. Not confident at all	0 (0.0%)	0 (0.0%)	1 (33.3%)	1	
2. Slightly confident	0 (7.7%)	0 (0.0%)	0 (0.0%)	1	
3. Moderately confident	1 (16.7%)	8 (47.1%)	0 (0.0%)	11	
4. Very confident	1 (16.7%)	4 (23.5%)	1 (33.3%)	9	
5. Extremely confident	4 (66.7%)	5 (29.4%)	1 (33.3%)	12	
Mean confidence (Mean ± SD)	4.50 ± 0.84	3.83 ± 0.92	3.25 ± 1.71		0.217
Resources desired					
More detailed instructions from my provider	1 (16.7%)	9 (37.5%)	1 (25.0%)	11	0.588
Videos or tutorials	1 (16.7%)	8 (33.3%)	1 (25.0%)	10	0.71
Access to a healthcare professional for questions	0 (0.0%)	3 (12.5%)	0 (0.0%)	3	0.50
No additional resources needed	4 (66.7%)	3 (12.5%)	2 (33.3%)	9	0.02*
Instruction source					
From my dentist or dental hygienist	4 (66.7%)	15 (62.5%)	2 (50.0%)	21	0.83
From my dentist or dental hygienist AND Printed materials provided by dental office	1 (16.7%)	1 (4.2%)	0 (0.0%)	2	0.44
I have not received specific instructions	1 (16.7%)	8 (33.3%)	2 (50.0%)	11	0.53
Challenges experienced					
Cost of specialized cleaning tools	0 (0.0%)	1 (4.2%)	0 (0.0%)	1	0.81
Difficulty accessing the implant area	1 (16.7%)	2 (8.3%)	1 (25.0%)	4	0.58
Time constraints	0 (0.0%)	1 (4.2%)	0 (0.0%)	1	0.81
Uncertainty about proper cleaning techniques	1 (16.7%)	7 (29.2%)	1 (25.0%)	9	0.82
Difficulty accessing the implant area AND Uncertainty about proper cleaning techniques	1 (16.7%)	1 (4.2%)	1 (25.0%)	3	0.30
No significant challenges	5 (83.3%)	14 (58.3%)	3 (75.0%)	22	0.47

* *p* <0.05.

### 3.6 Patient knowledge and awareness

Knowledge scores for three domains (implant disease risk factors, systemic health connections, and periodontitis relationship) are presented in Table 7. No significant differences in knowledge levels were demonstrated between diagnosis groups for any domain. When asked about understanding of infections related to dental implants, 38.2% reported full understanding, 32.4% reported being somewhat aware, and 29.4% reported no prior awareness of the relationship. Similarly, knowledge of systemic health conditions affecting implants and the relationship between periodontal health and peri-implant health did not differ significantly across groups. However, patients diagnosed with peri-implantitis demonstrated the lowest mean scores for systemic health knowledge compared to those with peri-implant health or peri-implant mucositis, although this difference did not reach statistical significance. When stratified by peri-implant diagnosis, of the ones with peri-implant health, only 33.3% of patients understood that implants could develop infections, 16.7% understood that systemic diseases could affect implant health, and 66.7% understood that having a history of periodontitis increases risk for developing peri-implant disease. Of the patients with peri-implantitis, 25% of patients understood that implants could develop infections, 25% understood that systemic diseases could affect implant health, and 50% understood that a history of periodontitis increases risk for developing peri-implant disease.

**Table 7:** Patient survey responses regarding knowledge-based domains (implant disease risk factors, systemic health connections, and periodontitis relationship), *n* (%).

Survey Statement	PI Health (*n* = 6)	PI Mucositis (*n* = 24)	Peri-implantitis (*n* = 4)	Total (*N* = 34)	*p*-value
Knowledge: Infections					
I fully understand that dental implants can develop infections similar to natural teeth	2 (33.3%)	10 (41.7%)	1 (25.0%)	13	0.79
I was somewhat aware that implants could get infected, but didn't know details	4 (66.7%)	5 (20.8%)	2 (50.0%)	11	0.07
I was/am not aware that implants could develop infections	0 (0.0%)	9 (37.5%)	1 (25.0%)	10	0.19
Knowledge: Systemic Health					
I understand that conditions like diabetes, smoking, or autoimmune disorders can affect my implant health	1 (16.7%)	13 (54.2%)	1 (25.0%)	15	0.18
I was aware of some connection but unsure of specific details	3 (50.0%)	3 (12.5%)	1 (25.0%)	7	0.12
I was informed about this relationship but need more information	0 (0.0%)	1 (4.2%)	0 (0.0%)	1	0.81
I was/am not aware that my general health could affect my dental implant	2 (33.3%)	7 (29.2%)	2 (50.0%)	11	0.71
Knowledge: Periodontal Health					
I understand that having gum disease around teeth puts you at a higher risk of developing implant disease	4 (66.7%)	14 (58.3%)	2 (50.0%)	20	0.87
I am aware of some connection but unsure of specific details	1 (16.7%)	5 (20.8%)	0 (0.0%)	6	0.60
I was informed about this relationship by my dentist/dental hygienist but need more information	0 (0.0%)	2 (8.3%)	0 (0.0%)	2	0.64
I was/am not aware that having a history of gum disease could affect my dental implant	1 (16.7%)	3 (12.5%)	2 (50.0%)	6	0.19

### 3.7 Psychosocial factors

Confidence in managing implant home care did not differ significantly by diagnosis group, as shown in Table 6. Mean confidence scores were 4.50 ± 0.84 for peri-implant health, 3.83 ± 0.92 for peri-implant mucositis, and 3.25 ± 1.71 for peri-implantitis. Additionally, confidence level did not demonstrate a significant correlation with any clinical parameter. Education level trended towards an association with peri-implant disease but was not statistically significant (*p* = 0.089). In patients with peri-implantitis, none had a college degree or higher, compared to 83.3% of patients with healthy implants and 54.2% of patients with implants diagnosed with peri-implant mucositis. Self-reported stress levels did not differ by diagnosis group and showed no correlation with clinical outcomes.

Interestingly, no subject in any group reported experiencing any clinical symptoms related to their dental implants (Q4), despite 82.4% having a clinical diagnosis of peri-implant disease. Confidence in oral home care also did not significantly correlate with any clinical parameter. Among subjects with peri-implant mucositis and peri-implantitis, 57.1% reported high confidence in their home care abilities, compared to 83.3% of those with peri-implant health (*p* = 0.22). This misalignment between self-perception of home care abilities and objective parameters represent a critical finding of this pilot study.

## 4 Discussion

### 4.1 Prevalence of peri-implant diseases

This cross-sectional study found a high burden of peri-implant disease among patients presenting to a university-based dental clinic, with 70.4% of assessed implants diagnosed with peri-implant mucositis (42/71; 59.2%) or peri-implantitis (8/71; 11.3%). These prevalence rates fall within the recent reported prevalence range for peri-implant diseases of 7%–85% for peri-implant mucositis and 0%–60% for peri-implantitis (17).

### 4.2 Oral hygiene instructions

In the present study, nearly one-third of patients reported receiving no implant-specific home-care instructions, and reported professional instruction was more common among patients with peri-implant health than among those with peri-implant disease (83.3% vs. 64.2%). While peri-implant mucositis is frequently reported in clinical populations, the high proportion of affected implants in this cohort reinforces that peri-implant inflammation is common in routine care settings and can occur even among patients who report regular home care (18–20). Similarly, Brunello et al. (19) found that most patients recalled receiving implant hygiene instruction (91.2%) and guidance on the importance of regular check-ups (91.6%), suggesting that oral hygiene instruction is often delivered and retained at a basic level; however, fewer patients reported supervised, hands-on use of cleaning devices in-office (40.4%) or receiving information about potential complications and failures before treatment (58.9%), indicating that reinforcement and skill-based training may be insufficient for consistent, effective day-to-day practice. Interestingly, the Brunello study (19) study population reported most patients receiving implant-specific hygiene instructions (91.2%) compared to 67.6% in the present study. This stark difference may be due to variability in study population, since the previously mentioned study was conducted in Italy, while this one was conducted in the United States. Regardless of this contrast, both studies highlight the insufficiency of instruction quality to patients, indicating a potentially universal overlooked aspect in clinical practice that warrants greater attention and deserves prioritization in both clinical practice and future research studies.

Further, Şahin (21) evaluated 144 implants using 2017 World Workshop/EFP guideline criteria and found that patient-reported oral hygiene habits and product use (including very low interdental cleaning around implant prostheses) were largely similar across peri-implant health, mucositis, and peri-implantitis groups, indicating limited discrimination of disease status by self-reported behaviors. The study also showed that knowledge did not reliably translate into better hygiene practices, supporting the need for clinicians to actively align patient awareness with observable, effective implant-specific plaque control behaviors (21).

### 4.3 Home-care frequency vs. effectiveness

All patients with peri-implant health reported cleaning their implant daily, and most participants reported moderate-to-high confidence in their home care regardless of disease status; nevertheless, peri-implant disease remained common (70.4%), signifying critical deficiencies in primary prevention of peri-implant disease. Together, these findings suggest that self-reported cleaning frequency and confidence are poor indicators of effective biofilm disruption around implants, where technique, interdental device selection, and access around restorative contours are likely more influential than frequency alone. Similarly, Cheung et al. (10) observed in a community-based general practice cohort that implants cleaned by brushing alone and those with detectable plaque/calculus were significantly more likely to exhibit peri-implant disease, and that prosthetic factors limiting cleaning accessibility reduced implant success. In our cohort, plaque index was strongly associated with peri-implant diagnosis (*p* = 0.011), reinforcing those objective measures of plaque accumulation, rather than self-reported adherence, aligned more closely with peri-implant diagnosis.

### 4.4 Home-care methods

Among cleaning methods, powered toothbrush use was associated with significantly lower plaque accumulation, supporting its recommendation in implant maintenance protocols. Studies comparing powered and mechanical toothbrushes in patients diagnosed with peri-implant health have demonstrated positive impacts on inflammation and plaque levels in both groups with no statistically significant differences (22, 23). On the other hand, randomized controlled trials comparing toothbrushes for treating peri-implant mucositis have demonstrated superior clinical outcomes with powered toothbrushes (24).

Interdental cleaning patterns identified in the present study reveal potential gaps in current practice. String floss was the most common interdental aid used (82.4%), while interdental brushes, which are often recommended for implant home care, demonstrated significantly low adoption (8.8%). A randomized controlled trial comparing waist-shaped interdental brushes with straight-shaped brushes found that the waist-shaped brushes were superior in plaque removal, particularly at line angle sites (25). The low utilization observed in the present study may be reflective of inadequate patient education, unfamiliarity with effective technique, or perceived difficulty of use.

Oral irrigation devices were moderately used (32.4%), suggesting some patients recognize the need for supplemental cleaning beyond brushing alone. A study with a 7-year follow-up demonstrated no statistically significant differences in PD or BOP with the addition of an oral irrigator to regular brushing and interdental aids (26). Another study evaluating effects of the addition of an oral irrigator or interdental brush demonstrated that both groups provided superior clinical outcomes compared to brushing alone in reducing peri-implant mucosal inflammation (27).

Another interesting finding was the usage of oral mouthrinse among groups in the present study. Zero percent of healthy patients, 20.8% of peri-implant mucositis patients, and 50.0% of peri-implantitis patients reported regular use of a mouthrinse. While this trend is notable, it is important to account for the small sample size, particularly with the peri-implantitis group. The observed pattern more likely reflects reverse causation, where patients experiencing inflammation initiate the use of a mouthrinse as a symptomatic response, rather than the mouthrinse itself contributing to disease pathogenesis. Alternatively, some patients may use an oral mouthrinse as a substitute for mechanical interdental cleaning, perceiving chemical antimicrobial activity as equivalent to mechanical biofilm disruption. Further research with larger cohorts and longitudinal designs is needed to clarify whether this observed pattern represents a marker of existing disease, a behavioral response to symptoms, or inadequate mechanical cleaning practices alone. Regardless of this, these findings emphasize that chemical adjuncts should not replace thorough mechanical biofilm disruption and removal as part of an implant home care protocol.

Furthermore, it important to note the finding that 32.4% of all patients surveyed reported receiving no professional instruction on implant-related home care methods, providing relative context for possible suboptimal cleaning methods reported by this group.

Lastly, confidence levels in home care abilities did not appear to correlate with any clinical parameter. Our results demonstrated that among subjects with implants diagnosed with disease, 57.1% reported high confidence (a score of at least 4/5) in their home care, compared to 83.3% of healthy patients. This presents perhaps an even more impactful finding of the present pilot study, which is that patient confidence in home care abilities and also the absence of symptoms are not necessarily reliable indicators of peri-implant health.

### 4.5 Risk indicators of peri-implant disease

The association between peri-implant disease and a history of periodontitis in our study (Fisher exact test PI vs. others: *p* = 0.029) is consistent with prior literature (17, 28, 29). In a 20-year longitudinal cohort study, Roccuzzo et al. (28) reported that patients with a history of periodontitis, particularly those who were non-compliant with individualized supportive periodontal care, had significantly higher odds of peri-implant mucositis and peri-implantitis, and that higher full-mouth plaque and bleeding scores further increased risk. Biologically, patients with periodontitis may be more susceptible due to a heightened inflammatory response, persistent challenges with plaque control, or residual periodontal pockets that can act as reservoirs for pathogenic biofilms. Together, these findings support risk-based implant maintenance planning, including shorter supportive care intervals and reinforced interdental cleaning instruction for patients with a history of periodontitis.

Gender was not significantly associated with peri-implant disease (*p* = 0.089), although all peri-implantitis cases occurred in males with a history of periodontitis. The 2025 AO/AAP systematic review and meta-analysis by Galarraga-Vinueza et al. (17) evaluated sex as a patient-related risk indicator and found no statistically significant pooled association between male sex and either peri-implant mucositis (effect summary OR ≈ 1.02) or peri-implantitis (effect summary OR ≈ 1.28), with substantial heterogeneity across studies (17). Our observed association, although in line with this review, may reflect sampling variability (few peri-implantitis cases) or residual confounding by established correlates (e.g., periodontitis history, plaque control, smoking, diabetes, alcohol use, and compliance with supportive care). Accordingly, the sex/gender finding should be interpreted cautiously and viewed as hypothesis-generating. Larger studies that adjust for these covariates and account for clustering of multiple implants within patients are needed to clarify whether sex modifies risk or is indirectly related to other behavioral and systemic factors not evaluated in this study.

### 4.6 Limitations

This preliminary, cross-sectional survey provides a single time-point description of peri-implant disease status and related patient factors, thus eliminating the possibility of making any direct conclusions or causal statements. The major limitation of this exploratory study is the small sample size. The peri-implantitis group, in particular, only consisted of 8 dental implants from 4 patients. Thus, any statistical findings for this group are not considered reliable and should be interpreted with caution. Furthermore, these patients were treated by multiple providers and were at different stages of treatment. Several questions relied on the patient's ability to recall certain pieces of information, leading to potential recall bias due to the nature of the study. Adherence to recommended implant maintenance intervals (e.g., patient compliance) could not be reliably evaluated because maintenance data were often missing or inconsistently recorded. The intrinsic limitation of this study is that stated home-care behaviors may differ from actual practices. Although the patient-reported data is limited due to this, the authors attempted to collect as many pieces of objective data as possible as part of a complete peri-implant examination. Results from this study should be interpreted cautiously and confirmed in larger, longitudinal studies in similar practice settings. However, these preliminary findings highlight the critical need for further research in patient education strategies regarding dental implant home care practices.

## Data Availability

The raw data supporting the conclusions of this article will be made available by the authors, without undue reservation.
