# Supplementary material for: Patient knowledge, home care practices, and peri-implant disease: a preliminary survey analysis of awareness gaps and cleaning behaviors
Source: Front Oral Health. 2026 Jun 15;7:1826003. doi: 10.3389/froh.2026.1826003 (PMC13311023; doi:10.3389/froh.2026.1826003)
Supplement: Supplementary file 1 [file DataSheet1.docx]

**Supplemental File 2. Implant Home Care Survey.**

**1. How often do you clean your implant site?**

- Daily
- Every other day
- Weekly
- Occasionally
- Rarely/Never

**For how long** (typically)? _____ min ______ sec

**2. What method(s) do you use to clean and maintain your implant area? Select all that apply.**

- Manual toothbrush
- Electric toothbrush
- Interproximal/interdental brush (eg, Proxibrush)
- Floss (string floss, eg Glide)
- Superfloss
- Water flosser/irrigator (eg, Waterpik)
- Mouthwash/mouthrinse
- Other (please specify): __________

**3. Have you experienced any of the following issues with your implant in the past month? Select all that apply.**

- Redness or swelling
- Pain or discomfort
- Bleeding
- Drainage or discharge (eg suppuration/pus)
- Itching or irritation
- None of the above

**4. On a scale of 1 to 5, how confident do you feel about managing your implant care at home?**

1 - Not confident at all

2 - Slightly confident

3 - Moderately confident

4 - Very confident

5 - Extremely confident

**5. What additional resources or support would be helpful for your implant home care? Select all that apply.**

- More detailed instructions from my provider
- Videos or tutorials
- Access to a healthcare professional for questions
- Support groups or forums
- Other (please specify): __________
- Not applicable

**6. Where did you primarily learn about how to care for your dental implant(s)?**

**Select all that apply.**

- From my dentist or dental hygienist
- Online resources
- Printed materials provided by dental office
- Family/friends with implants
- I have not received specific instructions
- Other (please specify): __________

**7. What challenges, if any, do you face in maintaining your dental implant(s)?**

**Select all that apply.**

- Difficulty accessing the implant area
- Uncertainty about proper cleaning techniques
- Time constraints
- Discomfort during cleaning
- Cost of specialized cleaning tools
- No significant challenges
- Other (please specify): __________________

**8. Which statement best describes your understanding of infections related to dental implants?**

- I fully understand that dental implants can develop infections similar to natural teeth
- I was somewhat aware that implants could get infected, but didn't know details
- I was/am not aware that implants could develop infections

**9. What is your understanding of the relationship between systemic conditions and implant health? (Select the most applicable statement)**

- I understand that conditions like diabetes, smoking, or autoimmune disorders can affect my implant health
- I was informed about this relationship but need more information
- I am aware of some connection but unsure of specific details
- I was/am not aware that my general health could affect my dental implant

**10. What is your understanding of the relationship between gum health and implant health? (Select the most applicable statement)**

- I understand that having gum disease around teeth puts you at higher risk of developing implant disease.
- I was informed about this relationship by my dentist/dental hygienist but need more information.
- I am aware of some connection but unsure of specific details.
- I was/am not aware that having a history of gum disease could affect my dental implant.

**11. Please select your education level:**

- Less than high school degree
- High school degree or equivalent
- Some college but no degree
- College degree or higher

**12. Considering the past month, please rate your stress level on a scale of 0-10:**

- 0-2: Minimal stress, feeling calm and in control
- 3-4: Mild stress, manageable daily pressures
- 5-6: Moderate stress, occasionally feeling overwhelmed
- 7-8: High stress, frequently feeling pressured or anxious
- 9-10: Severe stress, feeling overwhelmed most of the time

---

Thank you for participating in this survey. Your feedback is important to us in ensuring that all patients have the best possible experience with their implant care.
